# Getting TRAIL back on track for cancer therapy

**DOI:** 10.1038/cdd.2014.81

**Published:** 2014-06-20

**Authors:** J Lemke, S von Karstedt, J Zinngrebe, H Walczak

**Affiliations:** 1 Centre for Cell Death, Cancer and Inflammation (CCCI), UCL Cancer Institute, University College London, 72 Huntley Street, London WC1E 6DD, UK; 2 Clinic of General and Visceral Surgery, University of Ulm, Albert-Einstein-Allee 23, 89081 Ulm, Germany

## Abstract

Unlike other members of the TNF superfamily, the TNF-related apoptosis-inducing ligand (TRAIL, also known as Apo2L) possesses the unique capacity to induce apoptosis selectively in cancer cells *in vitro* and *in vivo*. This exciting discovery provided the basis for the development of TRAIL-receptor agonists (TRAs), which have demonstrated robust anticancer activity in a number of preclinical studies. Subsequently initiated clinical trials testing TRAs demonstrated, on the one hand, broad tolerability but revealed, on the other, that therapeutic benefit was rather limited. Several factors that are likely to account for TRAs' sobering clinical performance have since been identified. First, because of initial concerns over potential hepatotoxicity, TRAs with relatively weak agonistic activity were selected to enter clinical trials. Second, although TRAIL can induce apoptosis in several cancer cell lines, it has now emerged that many others, and importantly, most primary cancer cells are resistant to TRAIL monotherapy. Third, so far patients enrolled in TRA-employing clinical trials were not selected for likelihood of benefitting from a TRA-comprising therapy on the basis of a valid(ated) biomarker. This review summarizes and discusses the results achieved so far in TRA-employing clinical trials in the light of these three shortcomings. By integrating recent insight on apoptotic and non-apoptotic TRAIL signaling in cancer cells, we propose approaches to introduce novel, revised TRAIL-based therapeutic concepts into the cancer clinic. These include (i) the use of recently developed highly active TRAs, (ii) the addition of efficient, but cancer-cell-selective TRAIL-sensitizing agents to overcome TRAIL resistance and (iii) employing proteomic profiling to uncover resistance mechanisms. We envisage that this shall enable the design of effective TRA-comprising therapeutic concepts for individual cancer patients in the future.

## Facts

- The discovery that TRAIL can induce apoptosis selectively in cancer cells has initiated the development of TRAs for clinical application.
- First results of TRA-employing clinical trials revealed that TRAs are well tolerated but failed to show robust therapeutic activity in patients.
- In recent years, it has emerged that, although TRAIL can induce apoptosis in many cancer cell lines, many other cell lines and most primary cancers are TRAIL resistant. Noteworthy, in some TRAIL-resistant cancer cells TRAIL can trigger non-apoptotic signaling pathways, which can contribute to their malignancy.
- Several means to overcome TRAIL resistance of cancer cells have been reported, including combination of TRAs with chemo- or radiotherapy or with targeted small molecules such as Smac mimetics, BH3 mimetics or inhibitors of kinases or the proteasome.

## Open Questions

- Why could the promising preclinical results obtained with TRAs not be successfully translated into anticancer activity in patients?
- What are novel TRAs with improved pharmacokinetic properties which could be taken forward into clinical application without causing toxicity?
- Which TRAIL-apoptosis sensitizing strategies should be considered for novel therapeutic combinations to overcome TRAIL resistance?
- How can reliable molecular markers be identified to select cancer patients that are likely to benefit from particular TRA-comprising therapies?

Most current therapeutic strategies to treat cancer patients aim to overcome two key hallmarks of cancer, i.e., excessive proliferation and apoptosis resistance.^1^ In contrast to inhibiting proliferation, which will mostly achieve stable disease by limiting tumor outgrowth, induction of apoptosis bears the potential to eliminate cancer cells, which could provide an opportunity for cure.

Apoptosis can be induced via an intrinsic and an extrinsic pathway. The intrinsic apoptosis pathway senses cellular damage, including misbalanced intracellular homeostasis, oxidative stress and DNA damage, thereby triggering the cell death program to eliminate non-functional cells and to maintain tissue integrity.^2^ As this pathway depends on mitochondria, it is also referred to as the ‘mitochondrial' apoptosis pathway.^3^ A central molecule in detecting cellular damage and triggering intrinsic apoptosis is the tumor-suppressor protein p53. Importantly, most conventional radio- and chemotherapies induce DNA damage that activates the intrinsic apoptotic pathway in a p53-dependent manner.^4^ However, functional inactivation of p53, either by mutation or loss of expression, is one of the most common genetic events in cancer. Hence, most cancer cells are either primarily resistant or acquire resistance to these conventional therapies.^5^

The extrinsic apoptosis pathways are triggered by binding of death ligands to transmembrane receptors, so-called death receptors (DRs), thereby transmitting a death signal coming from the outside of the cell. Importantly, even though p53 has been shown to be capable of influencing DR-induced apoptosis signaling in certain cancers, mainly by influencing DR expression itself,^6, 7^ in most cases p53 appears to be dispensable for apoptosis induction by DRs.^8^ Hence, stimulation of the extrinsic apoptosis pathway is bound to be more effective than chemotherapy for treating cancers with *TP53* mutations.

Discovered in 1975, the first death ligand considered for anticancer therapy was tumor necrosis factor (TNF).^9^ However, although TNF induced cell death in some cancer cells, it soon became evident that TNF's primary function is the production of pro-inflammatory factors and that this activity is causative for the severe toxicity induced by systemic application of this cytokine.^10, 11, 12^ Subsequently, two agonistic antibodies targeting the newly identified DR FAS/APO-1 (CD95) were shown to be capable of inducing apoptosis in a wide range of cancer cells.^13, 14, 15, 16^ Again, initial optimism to target CD95 for anticancer therapy was stunted by the fact that systemic treatment with recombinant CD95L or CD95-agonistic antibodies resulted in fulminant and lethal hepatotoxicity.^17^ A few years later, TNF-related apoptosis-inducing ligand (TRAIL/Apo2L) was identified based on its sequence homology to TNF and CD95L.^18, 19^ Similar to TNF and CD95L, TRAIL induced apoptosis in cancer cells. Importantly, however, and in contrast to TNF and CD95L, systemic treatment with TRAIL *in vivo* killed tumor cells without causing toxicity.^20, 21^ Thereby, a death ligand with the promising feature of cancer selectivity had been discovered. Apart from sparking the development of TRAIL-receptor (TRAIL-R) agonists (TRAs) for clinical application as potential novel cancer therapeutics, this discovery resulted in intense world-wide research efforts to unravel the signal transduction machinery triggered by this ligand, especially concerning apoptosis induction in cancer cells and how resistance to TRAIL-induced apoptosis may be overcome when it is encountered.

## TRAIL-Induced Apoptosis

**Figure 1:**
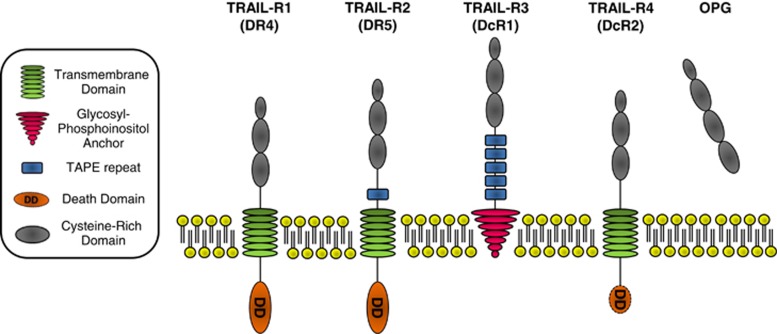
Overview of the TRAIL-R system in humans. TRAIL can bind to four membrane-bound and to one soluble receptor. TRAIL-R1 (DR4) and TRAIL-R2 (DR5) can induce apoptosis via their DDs. In contrast, TRAIL-R3 (DcR1), TRAIL-R4 (DcR2) and the soluble receptor osteoprotegerin (OPG) have been suggested to impair TRAIL-induced apoptosis as they are capable of binding to TRAIL but lack a functional DD required for apoptosis induction. TRAIL-R3 is as glycosyl-phosphatidyl-inositol-anchored protein that completely lacks an intracellular domain. TRAIL-R4 is inserted in the membrane via a transmembrane domain but only expresses a truncated death domain, which is incapable of inducing apoptosis

**Figure 2:**
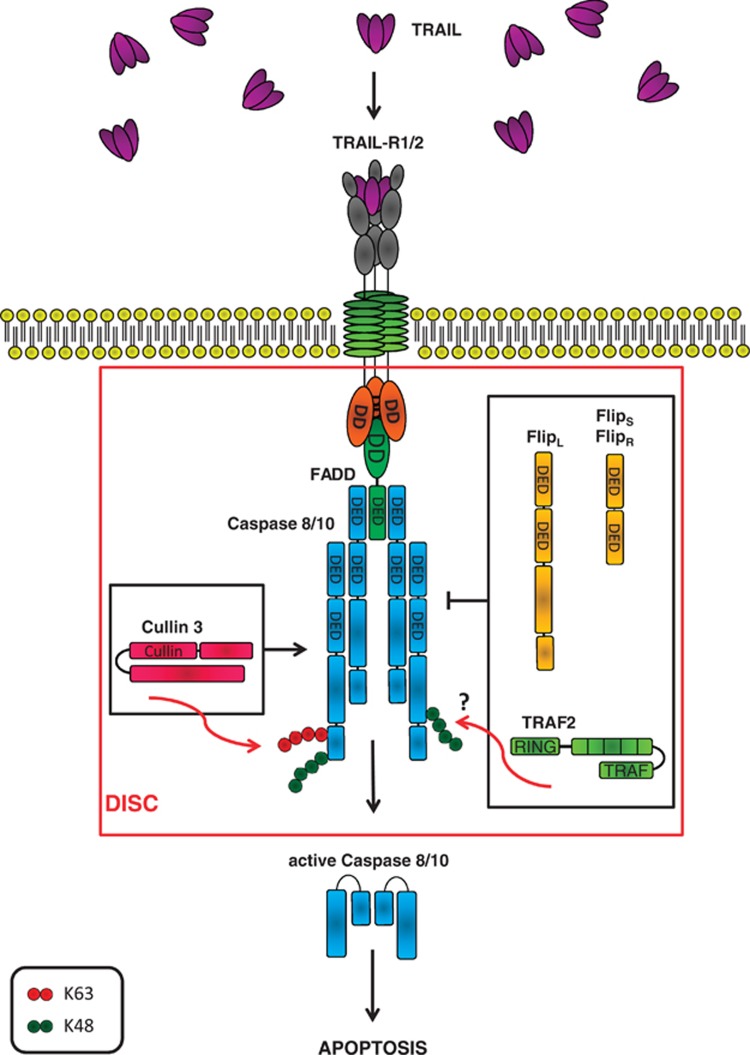
The current model of TRAIL-induced DISC formation. Upon binding of trimerized TRAIL to TRAIL-R1/2, the adaptor molecule FADD is recruited via homotypic DD interaction. Subsequently, FADD recruits pro-caspase-8/10 molecules via their respective DEDs. These pro-caspases are cleaved and activated at the DISC, initiating the apoptosis signaling cascade. The E3 ligase Cullin3 has been shown to stabilize DISC formation by polyubiquitination of caspase-8. Different forms of cFLIP can inhibit DISC formation by competing with caspase-8/10 for binding to FADD. TRAF2 has been suggested to negatively regulate DISC activity by promoting K48-linked ubiquitination and subsequent proteasomal degradation of caspase-8

Two TRAIL-Rs are capable of transmitting apoptosis, i.e., TRAIL-R1 (also known as DR4)^22^ and TRAIL-R2 (also known as Apo2, KILLER, DR5 or TRICK2; Figure 1).^7, 23, 24, 25, 26^ Binding of TRAIL, which naturally occurs as a trimer, to TRAIL-R1 and/or TRAIL-R2 induces receptor trimerization, the prerequisite for formation of the death-inducing signaling complex (DISC). The adaptor protein Fas-associated protein with death domain (FADD) is recruited to the death domain (DD) of these TRAIL-Rs via its own DD. FADD in turn recruits pro-caspase-8/10 to the DISC via homotypic death effector domain (DED) interaction as both FADD and these caspases contain DEDs capable of interacting with each other.^27, 28, 29, 30^ Both caspase-8 and caspase-10 are recruited to and activated at the DISC. Whereas caspase-8 is the apoptosis-initiating caspase at the DISC, caspase-10 is not required for apoptosis induction and indeed cannot substitute for caspase-8 as pro-apoptotic caspase at the DISC.^29^ Caspase-8 is recruited as an enzymatically inactive pro-caspase. It is activated by a proximity-induced conformational change at the DISC and subsequently fully activated by auto-catalytic cleavage and formation of homodimers (reviewed in Kantari and Walczak^31^). Upon release of active homodimers from the DISC, caspase-8 cleaves and activates downstream substrates of the apoptotic pathway (summarized in Figure 2). Recent work using quantitative mass spectrometry has shed light on the stoichiometry of the TRAIL-DISC, by demonstrating that three TRAIL-R1/2 receptors recruit only one FADD molecule, which subsequently recruits multiple pro-caspase-8 molecules.^32^ Based on the presence of two DEDs in caspase-8, the authors propose a model in which the first pro-caspase-8 protein is recruited to the DISC via interaction with the DED of FADD, whereas additional pro-caspase-8 molecules are recruited to the first one by interaction via their respective DEDs resulting in chain formation of pro-caspase-8 molecules. Intriguingly, a very similar model of DISC stoichiometry was also reported for the CD95-system.^33^

In addition to TRAIL-R1 and TRAIL-R2, TRAIL can also bind to two non-DD-containing membrane-bound receptors, TRAIL-R3 (also known as decoy receptor 1 (DcR1))^23, 25, 34, 35, 36^ and TRAIL-R4 (DcR2)^37, 38, 39^ (Figure 1). Although the extracellular domains of these receptors are highly homologous to those of TRAIL-R1/2, TRAIL-R3 is a glycosyl-phosphatidyl-inositol-anchored receptor lacking an intracellular domain and TRAIL-R4 only contains a truncated, non-functional DD in its intracellular domain. Consequently, these two receptors are incapable of inducing apoptosis. As TRAIL-R3/4 can nevertheless bind TRAIL, they might compete with the apoptosis-inducing DD-containing TRAIL-Rs for ligand binding, which led to the hypothesis that these receptors may act as decoys for TRAIL. Indeed, they were both shown to be capable of inhibiting TRAIL-induced apoptosis when overexpressed.^40, 41^ In addition to a possible TRAIL-sequestering function, TRAIL-R4 might impair TRAIL-induced apoptosis by forming inactive hetero-complexes with TRAIL-R2,^40, 42^ and/or by triggering anti-apoptotic signaling pathways such as NF-*κ*B and PKB/AKT.^37, 43^ Nevertheless, as all of the above-mentioned mechanisms have almost exclusively been studied in overexpression systems, the physiological function of endogenously expressed TRAIL-R3/4 and whether they can indeed interfere with TRAIL-induced apoptosis under physiological expression conditions remains to be established. Clinically, this understanding will be of great interest given that TRAIL-R3 is highly expressed in many primary gastrointestinal cancers^44^ and that high TRAIL-R4 expression is associated with poor prognosis in breast cancer.^45^

In addition to the four membrane-bound TRAIL-Rs, the soluble TNF receptor superfamily member Osteoprotegerin (OPG), mainly involved in regulating osteoclast activity by inhibiting RANKL,^46^ has also been reported to interact with TRAIL^47^ (Figure 1). Whereas exogenous application of recombinant OPG has indeed been shown to be capable of inhibiting TRAIL-induced apoptosis,^48, 49^ the apparently rather low affinity between TRAIL and OPG at physiological temperatures casts doubt on the physiological relevance of this interaction and, hence, of endogenously expressed OPG in impairing TRAIL signaling.^50^ This notion is further supported by the intriguing observation that no *in vivo* study in which high-dose TRAIL treatments have been employed over extended periods of time has reported any bone anomalies. This would, however, be expected in such animals if the TRAIL–OPG interaction were of physiological relevance. In summary, TRAIL is the most promiscuous TNF family member as it binds to at least four different receptors.

The physiological reason for having such a variety of TRAIL-Rs in humans is still not fully understood. Intriguingly, in mice only one death-inducing receptor is expressed, mTRAIL-R (MK/mDR5), which shares 43% and 49% sequence homology with human TRAIL-R1 and TRAIL-R2, respectively.^51^ In addition to mTRAIL-R, mice express two potential decoy receptors, mDcTRAIL-R1 and mDcTRAIL-R2, which are, however, quite distinct from the human decoy receptors, TRAIL-R3 and -4.^52^ These differences between mice and men suggest that the presence of two TRAIL-R genes with a full DD in humans might have been a rather recent evolutionary event, and it is still not entirely clear as to why humans have two DD-containing TRAIL-Rs.

### The emerging role of the ubiquitin-system in controlling DISC activity

Recent evidence suggests post-translational modification by ubiquitination to be a crucial regulator of DISC activity. The E3 ligase Cullin3 has been reported to be recruited to the DISC where it was shown to poly-ubiquitinate caspase-8, leading to DISC recruitment of the ubiquitin-binding protein p62, which stabilizes activated caspase-8, thereby facilitating DISC activation.^53^ Second, it has been shown that TNF receptor-associated factor 2 (TRAF2) is recruited to the DISC where it mediates attachment of K48-linked ubiquitin chains to caspase-8. This targets caspase-8 for proteasomal degradation and, hence, limits DISC activity.^54^ Whether or not this K48-linked ubiquitination is directly mediated by TRAF2 is currently debated as structural and functional studies by others concluded that TRAF2 may not be able to act as an E3 ligase on its own.^55, 56^ In summary, the DISC is a highly dynamic protein complex that requires tight regulation. This regulation is, at least in part, achieved by ubiquitination (Figure 2).

### Type-I *versus* type-II cells

**Figure 3:**
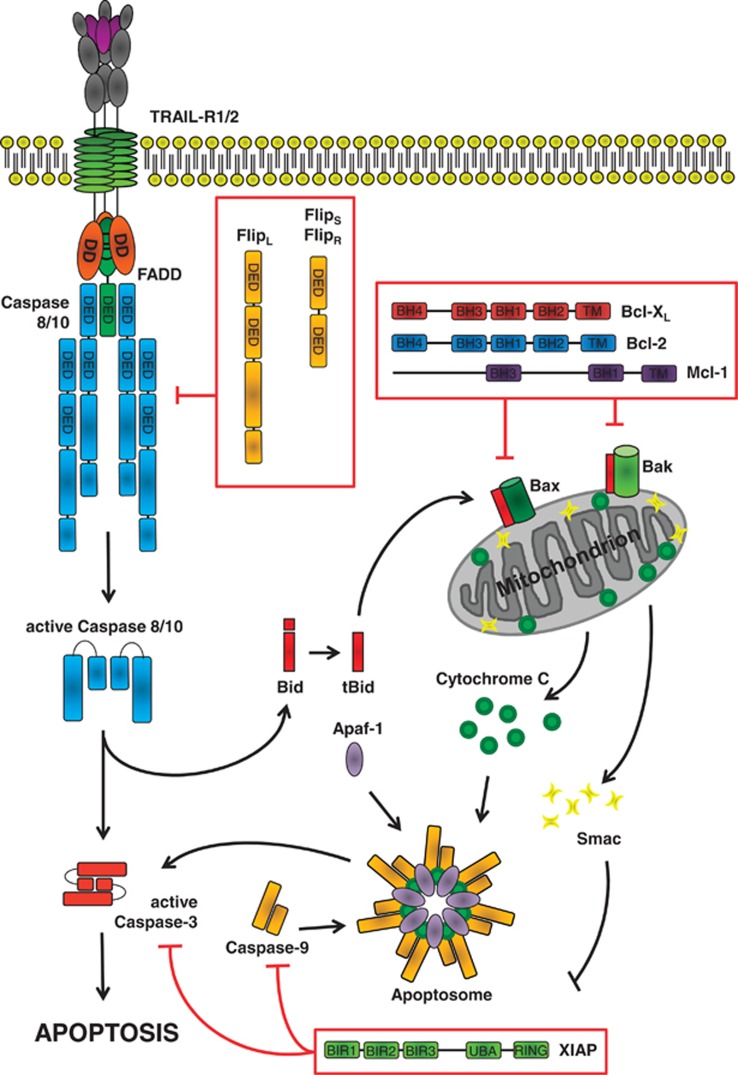
TRAIL-induced apoptosis. In type-I cells, DISC-activated caspase-8 is sufficient to directly cleave and activate the downstream effector caspase-3. In type-II cells, activation of the mitochondrial apoptosis pathway, mediated by caspase-8-dependent cleavage of Bid, is required to achieve effector caspase activation. Truncated Bid (tBid) translocates to the mitochondria where it induces Bax/Bak-mediated mitochondrial outer membrane permeabilization (MOMP). MOMP releases pro-apoptotic factors such as cytochrome-*c* and Smac/DIABLO into the cytosol. Cytosolic cytochrome-*c* aggregates with Apaf-1 and procaspase-9 to form the apoptosome, the activation platform for caspase-9. Active caspase-9 cleaves, and thereby activates, downstream effector caspases including caspase-3. The TRAIL-induced apoptosis cascade is inhibited at various levels: (i) at the DISC, cFLIP competes with caspase-8 for binding to FADD; (ii) at the mitochondria, anti-apoptotic Bcl-2 family members like Bcl-2, Bcl-xL and Mcl-1 inhibit pro-apoptotic Bax- and Bak-mediated MOMP and (iii) at the level of effector caspases, XIAP inhibits them by direct interaction. Hence, together with the extent of DISC-generated caspase-8 activity, the ratio of the expression of caspase-3 to XIAP is crucial for categorization of a particular cell line as type-I or type-II

Cells have been classified in two categories based on the pathway which they employ for apoptosis induction upon DISC activation, a phenomenon which was first described for the CD95/CD95L system.^57^ In so-called type-I cells, DISC activation is strong and stable enough to induce robust caspase-8 activation, which is, in turn, sufficient to directly and fully activate the effector caspase-3, resulting in apoptosis. In type-II cells, DISC-induced caspase-3 activation is incomplete and insufficient to induce apoptosis. Therefore, additional triggering of the mitochondrial pathway is required to induce apoptosis in these cells. To achieve triggering of the mitochondrial pathway, caspase-8 cleaves the pro-apoptotic BH3-only protein Bid, generating truncated Bid.^58^ Truncated Bid in turn activates the pro-apoptotic Bcl-2-family members Bax and Bak enabling them to permeabilize the mitochondrial outer membrane (reviewed in Westphal *et al*^59^). Upon mitochondrial outer membrane permeabilization (MOMP), pro-apoptotic factors such as cytochrome-*c* are released. Cytoplasmic cytochrome-*c* associates with Apaf-1 and pro-caspase-9 to form the multiprotein complex known as the apoptosome, an activation platform for pro-caspase-9 that initiates effector caspase cleavage ultimately leading to apoptosis (Figure 3).

Initially, differential efficiency in forming an active DISC was thought to be the decisive factor distinguishing type-I from type-II cells.^60^ However, more recently it was demonstrated that the anti-apoptotic factor X-linked inhibitor of apoptosis proteins (XIAPs) is a crucial factor in making this distinction.^61^ XIAP counteracts apoptosis induction by directly inhibiting caspase-3,^62^ and in type-II cells a high XIAP/caspase-3 ratio prevents full caspase-3 activation by caspase-8. In summary, it appears that the DISC's capacity to cleave caspase-3 and the ratio of XIAP to caspase-3 in a given cell are together critical for the distinction between type-I and type-II cells.

## TRAIL-Induced Necroptosis

Recently, it was demonstrated that DR triggering may also program cells to die in a caspase-independent, necrotic way (known as programmed necrosis or necroptosis).^63^ Necroptosis depends on the formation of a complex containing the kinases RIP1 and RIP3. This complex, also called necrosome, mainly forms in scenarios when caspase-8 is absent or when its activity is blocked. The necrosome in turn recruits and phosphorylates the pseudokinase MLKL, which was shown to be required for necroptosis induction.^64, 65^ How MLKL ultimately leads to necroptosis induction is still not fully understood, but recent studies suggest that MLKL might be recruited to the plasma membrane where it forms pores leading to membrane permeabilization.^66, 67^ Intriguingly, not only TNF but also CD95L and TRAIL were shown to be capable of inducing necroptosis.^68, 69, 70, 71^ Given the emerging role of necroptosis in pathophysiological processes and the resulting therapeutic potential of targeting necroptosis, this discovery may turn out to become important.

## Resistance Mechanisms to TRAIL-Induced Apoptosis

### Cellular FLICE-like inhibitory protein (cFLIP): regulator of caspase-8 activation at the DISC

To avoid excessive apoptosis induction by TRAIL, several mechanisms to countervail TRAIL-induced apoptosis have evolved in normal cells and are frequently exacerbated in tumor cells to escape TRAIL-induced apoptosis. Formation of the DISC is controlled by cFLIP (Figure 2). Three splice variants, cFLIP-long (cFLIP_L_), cFLIP-short (cFLIP_S_) and cFLIP-Raji (cFLIP_R_), are expressed on the protein level.^72, 73^ All three of these cFLIP variants contain two N-terminal DEDs that are highly homologous to the two DEDs of caspases-8 and -10. cFLIP_S_ and cFLIP_R_ are analogous as they both contain a short C-terminal domain, albeit slightly different ones. In contrast, cFLIP_L_ contains a long C-terminal domain, which closely resembles the caspase portion of caspase-8 but lacks catalytic activity. Both cFLIP_S_ and cFLIP_R_ compete with caspase-8 and casapse-10 for binding to FADD thereby inhibiting the pro-apoptotic activity of the DISC.^72, 73^ In contrast, the role of cFLIP_L_ in regulating apoptosis appears to be more complex and seems to depend on expression levels and the intensity of receptor stimulation (reviewed in Ozturk *et al*^74^). cFLIP_L_ acts similar to the short cFLIP variants in an anti-apoptotic manner when expressed at high levels. When expressed at lower levels, however, cFLIP_L_ can also facilitate apoptosis by enhancing pro-caspase-8 recruitment to the DISC.^75, 76^ Interestingly, cFLIP splice variants have also been shown to differentially control necroptosis induction. By completely preventing caspase-8 activation at the DISC, cFLIP_S_ can promote RIP-1/-3-dependent necroptosis induction. By contrast, cFLIP_L_ is thought to inhibit necroptosis in concert with caspase-8 because of formation of cFLIP_L_/caspase-8 heterodimers resulting in localized caspase-8 activity and consequent inactivation of RIP1 and RIP3.^71, 77, 78^ In summary, cFLIP is a crucial regulator of the DISC and can inhibit, promote or switch the signaling output of DR stimulation. Therefore, cFLIP is an attractive target when exploiting DR-induced cell death for cancer therapy.

### The Bcl-2 family: balancing death and survival at the mitochondrion

MOMP, the hallmark of the intrinsic apoptosis pathway, is positively and negatively regulated by three different classes of Bcl-2 family members: (i) pro-apoptotic effectors (Bax, Bak and possibly Bok), (ii) pro-survival factors (e.g., Bcl-2, Bcl-xL and Mcl-1) and (iii) pro-apoptotic inducers, which are BH3-only proteins (e.g., Bid, Bim, Puma, Noxa; reviewed in Shamas-Din *et al*^79^). The latter proteins are sensors for apoptotic stimuli and activated by transcriptional induction, posttranslational modification or, in case of Bid, by caspase-8-mediated proteolysis.^80^ Bax and Bak, the executioners of the mitochondrial apoptosis pathway, are kept in check by anti-apoptotic factors such as BcL-2, Bcl-xL and Mcl-1. Thus, the balance of pro- *versus* anti-apoptotic Bcl-2 family members tightly regulates MOMP and, thereby, the mitochondrial apoptosis pathway. Alterations in this balance, e.g., by overexpression of anti-apoptotic Bcl-2 family members or loss of expression of pro-apoptotic members, frequently occur in cancers with the consequence that these cancers are rendered resistant to conventional chemo- and radiotherapy (reviewed in Indran *et al*^81^). With respect to TRAIL signaling, it has been shown that type-II cancer cells can acquire resistance to TRAIL-induced apoptosis by loss of Bax^82^ or increased expression of Bcl-2,^83^ Bcl-xL^84^ or Mcl-1^85^ (Figure 3).

### Inhibitor of apoptosis proteins (IAPs): caspase inhibitors with emerging roles in the ubiquitin system

The family of IAPs comprises of a number of members of which XIAP, cellular IAP (cIAP)1 and cIAP2 have been most extensively studied. All IAPs are characterized by containing at least one baculovirus IAP repeat (BIR) domain. Their anti-apoptotic function has been known for a long time and has initially been solely attributed to their ability to directly inhibit caspases. Indeed, XIAP was shown to prevent activation of caspases-3, -7 and -9 via direct binding mediated by its BIR domains.^62^ As outlined above, its prominent role in regulating apoptosis is also highlighted by its critical function in distinguishing between type-I and type-II apoptosis signaling. In addition to cytochrome-*c*, MOMP also results in release of the second mitochondrial activator of caspases (Smac/DIABLO)^86^ from the mitochondrial intermembrane space into the cytosol. Cytosolic Smac directly binds to XIAP, thereby blocking its inhibitory function on effector caspases and, in turn, allowing for their full activation. Initially, the anti-apoptotic role of cIAP1 and cIAP2 was, similar to XIAP, mainly attributed to their direct inhibitory capacity toward caspases. Recently, however, it has emerged that, although cIAP1 and cIAP2 bind caspases, they cannot efficiently inhibit them.^87^ These results suggest that they exert their anti-apoptotic functions by other mechanisms than directly inhibiting caspases. Indeed, it has been demonstrated that cIAP1 and cIAP2, and also XIAP, possess E3-ligase activity via their Really Interesting New Gene (RING) domain, enabling them to ubiquitinate proteins.^88, 89, 90^ Besides auto-ubiquitination and degradation of cIAPs, several ubiquitination targets of cIAPs have been proposed, including caspase-3 and -7, targeting them for proteasomal degradation and thereby suppressing apoptosis.^91^ Furthermore, IAPs have been shown to also contribute to cell survival and apoptosis resistance in a caspase-independent manner by regulating a number of signaling pathways, most importantly NF-*κ*B signaling, by ubiquitination events (reviewed in Gyrd-Hansen and Meier^92^ and Silke and Meier^93^). In summary, IAPs are critical regulators of TRAIL-induced apoptosis by either directly inhibiting caspases, targeting caspases for proteasomal degradation and/or by regulating cell survival signaling pathways such as NF-*κ*B.

## Non-Cell Death Signaling Pathways Induced by TRAIL

In addition to apoptotic and in some cases necroptotic cell death, TRAIL treatment has been shown to induce a variety of non-cell death signaling pathways, including the NF-*κ*B, MAPK, Src and phosphoinositide 3-kinase pathways (reviewed in Azijli *et al*^94^). In line with the fact that activation of these pathways is known to promote malignancy of cancer cells, it could be demonstrated that TRAIL stimulation can enhance migration and invasion by activation of these pathways.^95, 96^ Moreover, *in vivo* TRAIL treatment led to enhanced metastasis in an orthotopic xenograft model of pancreatic cancer^96^ and TRAIL-R2 expression in the same model might promote tumor cell proliferation by suppressing maturation of the microRNA let-7.^97^ One interesting study has revealed that *KRAS-*mutated colorectal cancer cell lines are not only more resistant to TRAIL-induced apoptosis induction than KRAS wild-type cells, but instead are stimulated to migrate when treated with TRAIL.^98^ In summary, these findings highlight that treating certain TRAIL-resistant cancers with TRAs might even bear the unwanted risk of worsening disease burden. It is, therefore, imperative to understand non-apoptotic signaling in order to preempt it. Interestingly, many cancers highly express TRAIL-R1 and TRAIL-R2 and their expression is not commonly lost during cancer progression, suggesting that their endogenous expression might provide an, as of yet, unknown advantage for disease progression of certain cancers also during later stages. It will therefore be interesting to investigate the function of the endogenous TRAIL/TRAIL-R system in cancers that are resistant to its apoptotic signaling output in order to understand the full implication of high TRAIL-R expression in cancer biology.

## Clinical Testing of TRAs

**Figure 4:**
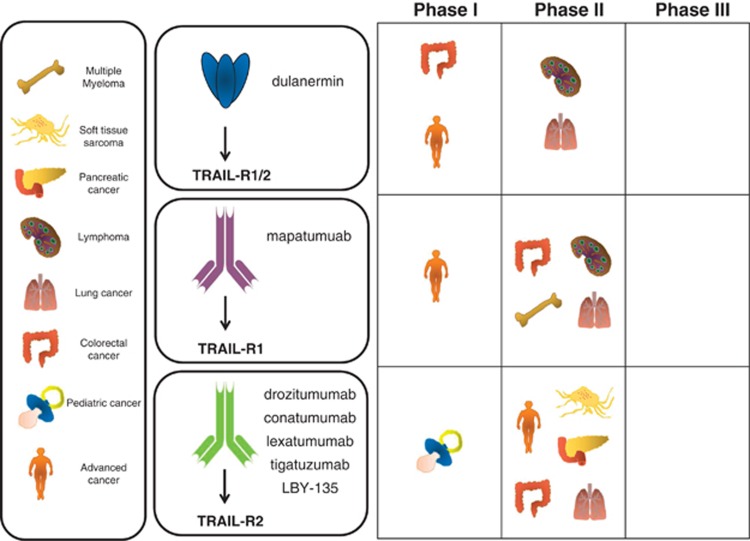
Progress of TRA in clinical trials. Schematic representation of the different TRA for which results of clinical trials have been reported. Cancer entities in which the different TRAs have been tested and the respective phase of clinical testing are shown

Initial optimism to utilize DRs for anticancer therapy was dampened by the fact that systemic application of TNF and CD95L provoked severe toxicity. Paradoxically, more than 20 years later, targeting the TNF/TNF-R and CD95/CD95L system has become attractive for cancer therapy again, however, with the opposing pharmacological concept, as blockade of TNF or appears to exert therapeutic benefit in certain malignancies.^99, 100^ In contrast, targeting of the TRAIL/TRAIL-R system has so far focused on inducing a death signal in tumor cells and different TRAs have been developed and already undergone first clinical testing (Figure 4).

### How to therapeutically target TRAIL-Rs: recombinant TRAIL *versus* agonistic TRAIL-R-specific antibodies

Current clinically tested TRAs comprise two categories of pharmacological agents: recombinant forms of TRAIL and agonistic antibodies specific for TRAIL-R1 or TRAIL-R2. Targeting both, TRAIL-R1 and TRAIL-R2, using recombinant TRAIL might be advantageous because triggering both death-inducing TRAIL-Rs at the same time might result in a stronger death signal than an agonistic antibody specific for only one receptor. However, TRAIL might also bind non-death-inducing TRAIL-Rs, which could hamper its apoptotic activity. Therefore, TRAIL-R1- or TRAIL-R2-specific TRAs such as antibodies would be more advised in the latter case. In this context, it is also interesting to note that for different cancer entities different contributions of TRAIL-R1 or TRAIL-R2 in transmitting TRAIL-induced signaling have been reported. Although colon and breast cancer cells primarily use TRAIL-R2 for apoptosis induction,^101^ lymphoid malignancies and pancreatic cancer cell lines have been reported to induce apoptosis primarily via TRAIL-R1.^102, 103, 104^ These differences in using one or the other receptor for apoptosis induction should be considered, when targeting cancer cells with TRAs specific for one of the two DD-containing TRAIL-R. In addition to these considerations, recombinant TRAIL and agonistic antibodies differ markedly in their pharmacokinetic properties. Whereas recombinant forms of human TRAIL are cleared within hours of systemic application, the half-life of therapeutic antibodies is typically in the range of several days to weeks. The higher half-life of antibodies hence circumvents the need for repeated or continuous application and allows a more stable concentration within cancerous tissues during treatment.

Surprisingly, it was recently shown that TRAIL treatment results in an antitumor effect in a mouse model in which tumor cells lack TRAIL-R expression suggesting that, at least in certain cases, TRAIL is also capable of exerting a therapeutic effect by targeting non-cancer cells, most likely cells in the tumor microenvironment. In the reported case, it was suggested that TRAIL-induced apoptosis in endothelial cells led to vascular disruption and tumor hemorrhage.^105^ This observation provides a novel approach to induce an antitumor effect by selectively targeting tumor vascularization. It should be noted, however, that tumor hemorrhage is a dreaded and potentially life-threatening event in cancer therapy and therefore patients should be carefully monitored for this potentially serious adverse effect during application of TRAs.

### Safety and anticancer activity of TRAs in clinical trials

Dulanermin, so far the only form of recombinant TRAIL developed for clinical application, comprises the TNF homology domain within the extracellular domain of human soluble TRAIL (amino acids 114–281).^20^ In a number of phase-I clinical trials, dulanermin was found to be safe and well tolerated even when combined with chemotherapy or the CD20-targeting antibody rituximab (Figure 4 and Table 1). Moreover, these studies revealed some antitumor activity evidenced by partial or complete clinical response in a subset of patients. However, to evaluate specific anticancer activity of novel therapeutic interventions randomized controlled trials (RCTs) are required in which patients are assigned into treatment groups to receive standard-of-care therapy alone or combined with (a) novel pharmacological compound(s). So far, dulanermin was evaluated in two RCTs: one in non-small-cell lung cancer comparing dulanermin/chemotherapy to chemotherapy alone^106^ and another in non-Hodgkin's lymphomas comparing dulanermin/rituximab to rituximab alone.^107^ Unfortunately, neither study revealed a significant anticancer activity that could have been attributed to dulanermin in these therapeutic regimes.

Apart from dulanermin, several agonistic TRAIL-R1- and TRAIL-R2-specific antibodies have entered clinical trials with one of them (mapatumumab) targeting TRAIL-R1 and all others (conatumumab, lexatumumab, tigatuzumab and drozitumab, LBY-135) targeting TRAIL-R2 (Figure 4 and Tables 2 and 3). For all of them, anticancer activity was demonstrated in preclinical models. Therefore, clinical trials were launched revealing safety and broad tolerability for all of them, both alone and in combination with standard therapy. For tigatuzumab the results of one, for mapatumumab the results of two, and for conatumumab the results of five, RCTs are available, which were conducted in soft tissue sarcoma, multiple myeloma, colorectal, pancreatic and lung cancer. All of these studies were carried out in combination with chemotherapy or the proteasome inhibitor bortezomib compared with the respective standard therapy alone (Tables 2 and 3). However, although some positive trends were observed, no statistically significant anticancer activity was achieved by addition of any of these TRAs in any RCT.

In summary, throughout all clinical studies dulanermin and all agonistic TRAIL-R antibodies were well tolerated, yet only minimal anticancer activity, which has not been confirmed in RCTs to date, was achieved. Thus, whereas toxicity of the evaluated TRAs is currently not to be expected, additional measures will have to be taken to achieve significant antitumor activity with TRAIL-based therapies.

## Future Direction to get TRAIL Back on Track for Cancer Therapy

Given the promising preclinical results, the failure of the TRAs that have thus far been tested clinically to exert robust anticancer activity in patients is disappointing. Nonetheless, if it were possible to identify the pitfalls of current TRA-based treatment approaches, it should be feasible to overcome these by novel strategies.

### Development of highly active TRAs

In addition to dulanermin, several other recombinant TRAIL preparations have been developed in which the amino terminus of the TNF homology domain of TRAIL was fused to tags such as poly-histidine (His), Flag, leucine Zipper (LZ) and isoleucine zipper (iz) tags. Although the first two tags merely serve a purpose in purification of the recombinant protein, addition of the LZ and iz sequence results in stabilization of TRAIL trimers via hydrophobic interactions within the trimerizing LZ and iz sequences.^21, 108^ Trimer stabilization results in increased agonistic activity of LZ- and iz-TRAIL as compared with other forms of TRAIL.^108^ Raising concern for some of these TRAIL variants, His-TRAIL and antibody-crosslinked Flag-TRAIL were shown to induce apoptosis in primary human hepatocytes *in vitro*,^108, 109^ whereas LZ- and iz-TRAIL were found to be non-toxic to human hepatocytes and can be safely applied *in vivo*.^21, 108, 110^ In conclusion, non-tagged soluble TRAIL has been shown to have the lowest antitumor efficiency, but on the other hand, also the lowest potential for toxicity. The latter was presumably one of the reasons why non-tagged recombinant TRAIL, dulanermin, was selected for clinical development.

Agonistic TRAIL-R antibodies are *per se* rather weak inducers of apoptosis, which is due to the fact that for efficient apoptosis induction via DRs their trimerization is required. The inherent bivalent nature of antibodies, however, only allows for crosslinking of two DRs. Thereby, only inefficient DISC formation is achieved, and further crosslinking of antibodies is required for efficient antibody-induced DISC formation.^111, 112^ This phenomenon has been intensively studied in the CD95 system since the early 1990s.^113^ Based on these considerations, the LZ- and iz-tagged forms of TRAIL were devised early on^21, 108^ but not considered for clinical development at the time for the above-mentioned concerns over potential toxicity of high-activity forms of recombinant TRAIL.

Yet, novel TRAs capable of forming stable higher-order complexes are currently developed (reviewed by Holland^114^). Among other approaches, TRAIL was fused to the Fc portion of human IgG1 (Fc-TRAIL), resulting in an increased capacity to oligomerize and a prolonged half-live *in vivo* as compared with soluble TRAIL. Importantly, Fc-TRAIL showed higher potency in inducing apoptosis in cancer cells *in vitro* and *in vivo* without exerting hepatotoxicity.^115^ Furthermore, a compound in which two trimers of the extracellular domain of TRAIL are fused to an Fc-part of human IgG1 generating a hexavalent TRA has recently been developed (APG350).^116^ APG350 demonstrated potent apoptosis induction in cancer cell lines, primary cancer cells and in xenograft mouse models. In addition, a novel tetrameric TRAIL-R2-activating nanobody, TAS266, has been developed for clinical use. TAS266 has been reported to be more effective than soluble TRAIL or agonistic antibodies *in vitro* and *in vivo*.^117^ However, it appears that a phase I clinical study with TAS266 in patients with advanced solid tumors was terminated early (clinicaltrials.gov). Although to date the reason for early termination of this study has not been disclosed, it raises concerns about the potential toxicity of this compound. In summary, first preclinical results obtained with these novel TRAs are promising, yet only clinical testing can ultimately reveal whether this promise will hold.

### Identification of potent and cancer-selective TRAIL sensitizers

#### Conventional chemotherapy and bortezomib in combination with TRAIL

The use of more active TRAs is likely to result in significantly enhanced activity against cancer cells that are susceptible to TRAIL-induced apoptosis. However, many cancer cells are intrinsically TRAIL resistant,^118^ indicating that crucial roadblocks in the form of resistance factors will need to be removed from the TRAIL apoptosis pathway in these cells in order to kill them by TRAIL. To date, a multitude of publications has demonstrated sensitization of cancer cell lines to TRAIL-induced apoptosis. However, many of these studies do not provide evidence for therapeutic activity *in vivo* and, thereby, also neglect potential toxicity. Thus, the use of such TRAIL-sensitizing strategies should be carefully evaluated for both efficiency and toxicity *in vivo* before consideration for clinical use.

Most commonly used standard chemotherapeutic agents including gemcitabine, irinotecan, doxorubicin, 5-FU and platinum-based agents such as cisplatin have been shown to synergize with TRAIL (reviewed in Newsom-Davis *et al*^119^). Various mechanisms have been proposed to underlie chemotherapy-induced TRAIL sensitization including increased DISC formation, upregulation of pro-apoptotic and suppression of anti-apoptotic proteins including of the pro- and potentially anti-apoptotic TRAIL-Rs. These findings provided the rationale to combine TRAs with conventional chemotherapy also in clinical trials. As explained above, none of the RCTs conducted to date, however, showed clinical activity attributable to the respective TRAs (Tables 1, 2 and 3).

Bortezomib, which is used in multiple myeloma treatment, shows antitumor activity alone and in combination with TRAs *in vitro* and/or *in vivo* in a broad range of cancers, including multiple myeloma, hepatocellular, breast, lung and pancreatic cancer (reviewed in de Wilt *et al*^120^). Yet, a recently completed RCT testing treatment of multiple myeloma with bortezomib as compared with bortezomib plus the TRAIL-R1-targeting TRA mapatumumab showed no additive therapeutic benefit.^121^ However, this may be due to the fact that mapatumumab bears the intrinsic crosslinking limitation of antibodies explained above, at least in the absence of sufficient Fc*γ* receptors in the vicinity of the tumor cells that could render this antibody active.^111, 112^ In summary, to date clinically available TRAs did not achieve additional clinical activity in combination with chemotherapy or bortezomib. Of note, the lack in efficacy of these combinations might be due to insufficient agonistic activity of the respective TRA employed, insufficient sensitization to TRAIL-induced apoptosis by the applied co-therapy, or indeed the combination of these two shortcomings. It will hence be interesting to test whether enhanced clinical activity can be achieved by combining potent TRAIL-sensitizing treatments with high-activity TRAs.

#### Smac und BH3 mimetics in combination with TRAIL

IAPs represent attractive targets for cancer therapy since high IAP expression is found in many cancers, which is associated with tumor progression and therapy resistance (reviewed by Fulda^122^). Hence, small molecules have been developed which mimic the XIAP-binding site of the endogenous XIAP-antagonist Smac, thereby antagonizing IAPs. This class of agents named Smac mimetics or IAP antagonists has shown promising preclinical activity alone or in combination with anticancer agents, which initiated the development of these small-molecule compounds for clinical application. Currently, a number of phase I and II clinical studies testing different Smac mimetic alone or in combination with chemotherapy are under way. Intriguingly, Smac mimetics have shown broad preclinical activity in sensitizing cancer cells to TRAIL-induced apoptosis *in vitro* and *in vivo* in a variety of cancer entities,^123, 124, 125, 126^ rendering them promising candidates for efficient TRA-comprising therapeutic approaches. Therefore, a clinical trial in ovarian cancer patients has been launched, which tests efficacy of the Smac mimetic birinapant in combination with the TRAIL-R2 agonist conatumumab (clinicaltrials.gov). Results of this trial will show whether the promising preclinical results will be translated into clinical activity.

As mentioned above, intrinsic resistance to the mitochondrial apoptosis pathway caused by high expression of anti-apoptotic Bcl-2 family members, such as Bcl-2, Bcl-xL and Mcl-1, is a common feature of cancer cells and associated with chemoresistance. To overcome mitochondrial apoptosis resistance, BH3 mimetics have been developed to antagonize anti-apoptotic Bcl-2 family members. To date, two such compounds have been evaluated in the clinic: ABT-199, which specifically targets Bcl-2, and ABT-263 (Navitoclax), which antagonizes both Bcl-2 and Bcl-xL.^127, 128^ BH3 mimetics have demonstrated impressive clinical activity in single therapy, and recently Bcl-2/xL inhibition by ABT-263, or its non-orally available analog ABT-737, has been shown to sensitize cancer cells to TRAIL *in vitro*.^129, 130, 131^ Thus, BH3 and Smac mimetics can be used to sensitize cancer cells to TRAIL-induced apoptosis by selectively inhibiting important roadblocks that obstruct the TRAIL apoptosis pathway.

#### Cyclin-dependent kinase 9 (CDK9) inhibition and TRAIL

Small-molecule inhibitors of several kinases are an emerging class of cancer drugs based on the fact that cancer cells have elevated kinase activity to enhance proliferation, migration and invasion, but also to maintain apoptosis resistance.^132^ We recently discovered selective inhibition of CDK9^133^ as the most powerful approach to overcome TRAIL resistance of cancer cells that we have come across in more than a decade of searching for potent TRAIL-sensitizing strategies.^110^ Intriguingly, CDK9 inhibition sensitizes cancer cells irrespective of their p53 status and can be applied together with a highly active recombinant form of TRAIL (iz-TRAIL) without causing hepatotoxicity within a considerable therapeutic window. Furthermore, the potency of this novel combination was underlined by the fact that it eradicated established orthotopic lung tumors *in vivo*. Mechanistically, CDK9 inhibition led to concomitant downregulation of cFLIP and Mcl-1 and, together, these two events were both required and sufficient for CDK9 inhibition-mediated TRAIL sensitization. The therapeutic principle that emerges from these findings is that *simultaneous* removal of multiple resistance factors is required to overcome TRAIL resistance and to efficiently kill cancer cells by TRAIL-comprising therapeutic combinations. This concept should be taken into account when devising future TRAIL-based cancer therapies.

In summary, besides conventional chemotherapy and proteasome inhibition, several novel, more-selective pharmacological approaches are at hand, which exert promising preclinical activity in combination with TRAIL. For the establishment of an active but non-toxic TRAIL-based therapeutic regime it will be decisive to evaluate these different compounds, also in combination, in conjunction with second-generation TRAs that exhibit higher agonistic activity than their predecessors.

### Identification of biomarkers that predict sensitivity to novel TRAIL-based therapies

It is interesting to note that among the majority of non-responding patients, in a subset of patients, TRAs showed signs of therapeutic benefit. These included two patients with chondrosarcoma, a cancer entity which is known to be largely resistant to conventional therapies. Intriguingly, both patients showed an antitumor response upon dulanermin monotherapy and in one of them long-term survival was achieved by combining surgery and prolonged dulanermin treatment.^134, 135^ These case reports reveal that recombinant forms of TRAIL, and possibly other TRAs, can be beneficial for individual patients even without additional sensitization strategies. However, so far it remains widely unresolved which markers, in addition to TRAIL-R1/2 expression, would identify and allow selection of patients that are likely to benefit from a TRA-comprising therapy. One report identified high expression of the O-glycosylation enzyme GALNT14 as a signature of TRAIL sensitivity in cancer cells. Mechanistically, the study showed that glycosylation of TRAIL-R2 leads to enhanced ligand-induced receptor clustering, facilitated DISC formation and subsequent apoptosis induction.^136^ Nevertheless, the value of using GALNT14 expression for predicting TRAIL sensitivity in patients remains to be shown as increased GALNT14 expression did not appear to significantly correlate with clinical response to dulanermin in a clinical study.^106^ In another approach, cell lines were systematically screened for responsiveness to TRAIL and, in parallel, expression of a panel of factors involved in executing or inhibiting the extrinsic apoptosis pathway was quantified. Data extracted from these results enabled predicting responsiveness to TRAIL. Possibly even more interestingly from a clinical perspective, the authors could utilize their system to propose a case-specific TRAIL-sensitizing strategy by analyzing the expression profiles from individual cell lines.^137^ Although these findings will require further validation in primary cancer cells and *in vivo*, this new and promising approach could prove to be valuable, given the recent advances in quantitative proteomics, which could enable the determination of protein expression profiles from patient-derived cancer tissue.

## Conclusion and Outlook

At the turn of the millennium, the discovery of TRAIL and its capacity to induce apoptosis selectively in tumor cells sparked the development of TRAIL and other TRAs as potential novel cancer drugs. Since then, the TRAIL signaling cascade has been the subject of intense research. However, 15 years on, first-generation TRAs could not live up to high expectations in clinical trials. The possible pitfalls have been identified, as outlined above, and strategies to tackle these are intensively investigated. Novel TRAs with increased agonistic properties are currently developed and high-potency, cancer-selective TRAIL sensitizers are appearing at the horizon. It is important to note that all preclinical *in-vivo* studies preceding the decision to start clinical trials with current TRAs were performed in xenograft or syngeneic graft mouse models. At the time, these were the standard mouse models available and have been helpful in evaluating preclinical activity of many anticancer therapies in the past. It has, however, become evident that these models lack many crucial aspects of the systemic disease that is cancer. This shortcoming has been addressed by the development of genetically engineered mouse models of various cancers, which closely resemble the full clinical spectra of the respective human cancers.^138, 139^ Hence, the challenge now is to determine whether newly devised high-activity TRAs combined with the most potent TRAIL-sensitizing strategies exert a therapeutic effect in these sophisticated mouse models of cancer to ultimately get TRAIL back on track to the cancer clinic.

## Figures and Tables

**Table 1:** Results of dulanermin (recombinant, soluble TRAIL) in clinical trials

**Dulanermin (AMG-951/rhApo2L)**						
**Phase**	***n***	**Cancer**	**Combination**	**Safety**	**Efficacy**	**Reference**
I	71	Advanced cancers	—	Safe	2 Responses (2PR)	^135^
I	23	Colorectal	Chemo+BV	Safe	13 Responses (13PR)	^140^
I	27	Colorectal	Chemo+BV	Safe	6 Responses (6PR)	^141^
I	30	Colorectal	Chemo+CX	Safe	NA	^142^
I	24	Lung	Chemo+BV	Safe	14 Responses (1CR/13PR)	^143^
I	7	Lymphoma	Rituximab	Safe	3 Responses (2CR/1PR)	^144^
II (RCT)	213	Lung	Chemo+BV	Safe	No anticancer activity^a^	^106^
II (RCT)	48	Lymphoma	Rituximab	Safe	No anticancer activity^a^	^107^

Abbreviations: BV, bevacizumab; chemo, chemotherapy; CR, complete response; CX, cetuximab; PR, partial response; *n*, number of patients enrolled; NA, data about responses (efficacy) were not reported; RCT, randomized-controlled trials

a Anticancer activity was considered when the addition of the TRA demonstrated statistically significant activity compared with the standard therapy

**Table 2:** Results of mapatumumab, an agonistic antibodies targeting TRAIL-R1 in clinical trials

**Mapatumumab (HGS-ETR1)**						
**Phase**	***n***	**Cancer**	**Combination**	**Safety**	**Efficacy**	**Reference**
I	49	Advanced cancers	—	Safe	No responses	^145^
I	41	Advanced cancers	—	Safe	No responses	^146^
I	49	Advanced cancers	Chemo	Safe	12 Responses (12PR)	^147^
I	27	Advanced cancers	Chemo	Safe	5 Responses (5PR)	^148^
I/II	40	Lymphoma	—	Safe	3 Responses (2CR/1PR)	^149^
II	38	Colorectal	—	Safe	No responses	^150^
II	32	Lung	—	Safe	No responses	^151^
II (RCT)	104	Multiple myeloma	Bortezomib	Safe	No anticancer activity^a^	^121^
II (RCT)	109	Lung	Chemo	Safe	No anticancer activity^a^	^152^

Abbreviations: chemo, chemotherapy; CR, complete response; *n*, number of patients enrolled; PR, partial response; RCT, randomized-controlled trial

a Anticancer activity was considered when the addition of the TRA demonstrated statistically significant activity compared with the standard therapy

**Table 3:** Results of TRAIL-R2-targeting agonistic antibodies in clinical trials

**Phase**	***n***	**Cancer**	**Combination**	**Safety**	**Efficacy**	**Reference**
*Conatumumab (AMG-655)*						
I	37	Advanced cancers	—	Safe	1 Response (1PR)	^153^
I	18	Advanced cancer	—	Safe	No responses	^154^
I	6	Soft tissue sarcoma	Chemo	Safe	No responses	^155^
I	9	Advanced cancers	Ganitumab	Safe	No responses	^156^
I	12	Lung	Chemo	Safe	4 Responses (1CR/3PR)	^157^
I	12	Colorectal	Chemo	Safe	5 Responses (5PR)	^158^
I	13	Pancreatic	Chemo	Safe	4 Responses (4PR)	^159^
II (RCT)	128	Soft tissue sarcoma	Chemo	Safe	No anticancer activity^a^	^155^
II (RCT)	172	Lung	Chemo	Safe	No anticancer activity^a^	^160^
II (RCT)	83	Pancreatic	Chemo	Safe	No anticancer activity^a^	^161^
II (RCT)	103	Colorectal	Chemo	Safe	No anticancer activity^a^	^162^
II (RCT)	190	Colorectal	Chemo+BV	Safe	No anticancer activity^a^	^163^
*Lexatumumab (HGS-ETR2)*						
I	37	Advanced cancers	—	Safe	No responses	^164^
I	31	Advanced cancers	—	Safe	No responses	^165^
I	41	Advanced Cancers	Chemo	Safe	Partial responses	^166^
I	24	Pediatric cancers	—	Safe	No responses	^167^
*Tigatuzumab (CS-1008)*						
I	17	Carcinoma or lymphoma	—	Safe	No responses	^168^
II	61	Pancreatic	Chemo	Safe	8 Responses (8PR)	^169^
II (RCT)	97	Lung	Chemo	Safe	No anticancer activity^a^	^170^
*Drozitumab (PRO95780/apomap)*						
I	9	Colorectal	Chemo	Safe	2 Responses (2PR)	^171^
I	50	Advanced cancers	—	Safe	No responses	^172^
*LBY-135*						
I/II	73	Advanced cancers	Chemo	Safe	2 Responses (2PR)	^173^

Abbreviations: BV, bevacizumab; chemo, chemotherapy; CR, complete response; *n*, number of patients enrolled; PR, partial response; RCT, randomized-controlled trial

a Anticancer activity was considered when the addition of the TRA demonstrated statistically significant activity compared with standard therapy

## Abbreviations

CDK: Cyclin-dependent kinase
cFLIP: cellular FLICE-like inhibitory protein
cIAP: cellular IAP
DD: death domain
DED: death effector domain
DR: death receptor
DISC: death-inducing signaling complex
FADD: Fas-associated protein with death domain
IAP: Inhibitor of apoptosis protein
iz: isoleucine zipper
LZ: leucine zipper
MOMP: mitochondrial outer membrane permeabilization
OPG: osteoprotegerin
RCT: randomized controlled trial
Smac: second mitochondrial activator of caspases
TNF: tumor necrosis factor
TRAF2: TNF-receptor associated factor 2
TRAIL: tumor necrosis factor-related apoptosis-inducing ligand
TRAIL-R: TRAIL-receptor
TRA: TRAIL-R agonist
XIAP: X-linked inhibitor of apoptosis protein

## Notes

HW is a named inventor on the patent that formed the basis for the development of Conatumumab. HW is cofounder, scientific advisor and shareholder of Apogenix GmbH (Heidelberg, Germany), a company that develops apoptosis-based drugs, including the TRAIL-R agonist APG350. The remaining authors declare no conflict of interest.
